# The First Persons Vaccinated in America

**Published:** 1869-03

**Authors:** 


The First Persons Vaccinated in America.—In July, 1800, Dr. Benjamin Waterhouse, of Cambridge, Mass., submitted four of his children to the new process, and they were the first persons vaccinated.

